# On the Clinical and Bacteriological Aspect of Mixed or Concurrent Infection in Pulmonary Tuberculosis1President’s address before the Central New York Medical Association, Buffalo N Y., October 16, 1894.

**Published:** 1894-11

**Authors:** Henry L. Elsner

**Affiliations:** Professor of the Science and Art of Medicine and Clinical Medicine, Syracuse Medical College


### ON THE CLINICAL AND BACTERIOLOGICAL ASPECT OF MIXED OR CONCURRENT INFECTION IN PULMONARY TUBERCULOSIS.[1]

BY HENRY L. ELSNER, M.D.,
Professor of the Science and Art of Medicine and Clinical Medicine, Syracuse Medical College.

THE privilege of presiding over your deliberations is at once an honor and a pleasure which I fully appreciate, and for which I thank you. Let us hope that the results of our coöperation today may redound to the credit of our profession; that we may return to our homes with enriched knowledge and the laudable ambition to renew the contest against disease with increased energy.

The subject which I purpose to bring to your notice today has been chosen after much thought and a thorough appreciation of the great responsibility devolving upon me. I have purposely chosen to unfold the Clinical and Bacteriological Aspect of Mixed Infection in Pulmonary Tuberculosis, for the reason that I wish to incite in my auditors, and in those to whom these words may be addressed ultimately, a desire to combine such methods of precision as have been made available for the more positive recognition and the consequent rational treatment of disease. It is true that the pathology and bacteriology of the subject has been carefully and scientifically investigated during the past decade, while the clinical picture has remained incomplete, and deserves to be brought before the profession for a more thorough discussion than it has yet received, when the various types of concurrent infection in acute and subacute tubercular phthisis shall be readily recognised and their significance better understood. In considering the clinical aspect of mixed infection in phthisis, I shall confine myself particularly to a class of cases in which bacteriological examination in conjunction with physical manifestations has made the coëxistence of an acute pneumonia with either a latent or active tuberculous process positive; a complex of disease which involves, as a rule, a large area of lung tissue, in which pathologic examination shows the presence of both processes mentioned, with one or the other in the ascendancy. The clinical picture is one of profound infection, terminating in a relatively short time; or if tuberculosis has preëxisted, the fatal termination is hastened by such concurrent infection. Andral[2] long ago called attention to the

1. President's address before the Central New York Medical Association, Buffalo N. Y., October 16, 1894.

2. Andral. *Clinique Medicale*, fourth edition, T. II., p. 36, ff.

occasional development of a croupous pneumonia in a tuberculous lung. In such cases, it was held that the tubercular disease antedating the pneumonia, was lighted to rapid degeneration by the addition of the pneumonic process. These were characterized by Morton[1] as "phthisis, a peri-pneumonia." In another class of cases it was supposed that the pneumonia developed in previously normal lungs, but that tuberculization ultimately followed.

In such cases resolution had been faulty, or it did not take place at all; dulness, flatness and bronchial breathing and crepitant with other râles, persisted, and finally tuberculosis developed in a lung, the seat of chronic inflammatory thickening. The form of pneumonia, according to Andral, which led to this condition, was the croupous. Herein lies the fault of Andral's reasoning, according to more recent authorities (Fränkel and Troje)[2], for, in these cases, there is no true pleuro-pneumonia which undergoes tuberculization, but the affection which gives rise to the symptoms is entirely different, both anatomically and clinically, certainly etiologically, a process which Virchow has called "smooth or gelatinous pneumonia. According to the writer's observation, it would be an absolute impossibility to state with any degree of certainty that a tuberculous herd was not already present in the lung or bronchial glands before the advent of the process which so closely simulated croupous pneumonia. Stokes[3], in speaking of the three forms in which acute tuberculosis shows itself, after considering first the ordinary miliary variety, speaks of a second form which probably corresponds to what, with our more recent methods as aids in diagnosis, we would class as mixed infection, and which he considered pneumonic owing to a continuation of the crepitant râles. The third variety was associated with hemoptysis, probably broncho-pneumonic, and corresponds closely with the cases which Baumler[4] has recently described so well, dwelling forcibly on the aspiration of infectious agents as a pathogenic factor. To this point the writer will again revert when considering the various forms of mixed infection in phthisis. Traube[5] was the first to elucidate the clinical picture of acute cheesy pneumonia. Possibly

1. Morton. Andral, I. c., T. I., p. 389.

2. Fränkel and Troje. *Zeitschrift für Klin. Medizin*, S. 33, Bd. 24.

3. Stokes. *Diagnose und Behandlung der Brust Krankheiten.* Translated by Busch, Bremen, 1838, p. 736, ff.

4. Baumler. *Deutsche Medizinische Wochenschrift*, 1893, p. i., etc.

5. Traube. *Uebergruene Sputa. Beitrage zur Pathologie und Physiologie, Bd. II., S.* 699-716.

some of his cases were of a croupous variety, concurrent with pulmonary tuberculosis; this last statement is justified when we take into consideration the fact that the cases which he describes could not be differentiated from ordinary fibrinous pneumonia during life. Large areas of lung tissue were involved or an entire lung was infiltrated. In many of these cases there was a "green sputum," which served to give the title to Traube's original paper. Such sputum, we now understand, is often found in ordinary croupous pneumonia, is almost translucent and glassy, occasionally of a grass-green color. The color depends on a modification of the blood-coloring matter, the presence of which is made certain by the large number of red blood-corpuscles found microscopically. These cases it would be difficult for us to classify. Traube, who lived before the day of bacteriological diagnosis, gave no data sufficiently scientific to justify us in concluding to which category his various cases belonged. His acute clinical observation, however, taught him to conclude that the cases of cheesy pneumonia were not ushered in with the decided chills so characteristic of ordinary croupous pneumonia, even when concurrent with acute pulmonary tuberculosis. He also called attention to the fact that in tuberculous pneumonia, bronchial breathing appears very late, sometimes as late as the end of the second week, the râles are large and coarse, rarely the characteristic crepitant râle of fibrinous pneumonia. These differential points, it is well for us to remember at this point, I shall have occasion to emphasize as differential aids before ending the discussion of this subject.

Walshe[1] believed that "pneumonia frequently occurs in the course of tuberculous disease or at its close. In the former case, it is either a mere local effect of the progress of tuberculization, or it may be extensive and acquire almost the importance of an idiopathic attack of the disease. But, even then, it is rarely of serious augury; it is, singularly enough, less fatal than primary pneumonia. Some of the most marked examples of rapid resolution I have met with were in phthisical persons. The mean duration of the inflammation even is less than when occurring in sound lungs." This latter observation is not, as a rule, confirmed by our clinical experience, though, in some cases, the resolution in concurrent catarrhal and croupous pneumonic areas has been rapid and satisfactory. The fact cannot be denied that, in this country, pneumonia accompanying the closing stage of tuberculous phthisis

1. Walshe. Disease of Lung, fourth edition, p. 442.

is almost invariably fatal. Walshe still further emphasizes the fact that, while pneumonia of the apex is not always tuberculous, it is, nevertheless, in the large majority of cases, when limited to the apex anteriorly, of tuberculous origin.

These data, coming to us from a time which antedates bacteriological diagnosis, while of great value owing to the high standing of the authorities quoted, and the fact that many were prompted by the results of authentic *post mortem* investigation, are certainly less convincing than the conclusions which it is my privilege to offer as the result of clinical observation, positive bacteriological evidence of the presence of two or more infectious agents, and ultimate anatomical examination.

What do we include under the head of mixed or concurrent infection in pulmonary tuberculosis? Those cases of tuberculosis, which are accompanied by inflammatory processes in the lung, caused by the concurrence of two or more infectious agents, one of which is the tubercle bacillus.

This, as I will explain later, allows of the consideration, in conjunction with this subject, of acute catarrhal and croupous pneumonia as concurrent with pulmonary tuberculosis, and those forms of pneumonic tuberculosis, in which the addition of streptococcus, or staphylococcus, to the tubercle bacillus cause the extension of the infiltration and exudation. This admixture finally hastens very materially the progress of the original active or latent disease—the latter being kindled into activity. In conjunction with this subject it will be wise for us to pause before proceeding further, to consider the results of mixed infection and determine whether we have clinical or bacteriological data at the present time, which justify us in concluding that the tubercle bacillus alone, without the presence of other infecting agents, has the power of causing changes in the lung which simulate very closely the various forms of acute and non-tuberculous pneumonia.

This question can be answered, I think, in the affirmative. My position is strengthened by the positive fact that, in accordance with advanced pathological as well as bacteriological research, which must of necessity influence us in our clinical studies of these cases, both lobular and diffuse desquamative pneumonia are the direct products of tubercular infection. Prudden,[1] in a recent experimental study on Concurrent Infection and the Formation of Cavities in Acute Pulmonary Tuberculosis, concludes that "the

1. Prudden. *New York Medical Journal*, July 7, 1894.

effects which may be induced in the living body by the tubercle bacillus are cell proliferation, productive and exudative inflammation, local obliteration of blood-vessels, necrosis and septic intoxication. These effects may all vary within wide limits, with the virulence of the infecting germs and the susceptibility of the affected individual. . . . The experimental studies on the tubercle bacillus and its effects on the living lung tissues of animals, soon make it clear that the tubercle bacillus cannot only cause the formation in the lungs of the new organized tissue, which in its various forms and marked tendency to cheesy degeneration we are wont to call tubercular, but also that it may, to a certain extent at least, be responsible for a simple exudating pneumonia, in no wise morphologically characteristic, and for a large proliferation of vesicular epithelium."

Clinically corresponding cases in the human subject are differentiated with great difficulty from the various forms of acute catarrhal and other forms of non-tubercular pneumonia. The previous history, with repeated microscopic examination of the sputum centrifugalized, can alone establish a positive diagnosis in many of these cases. The conclusion must be reached that the tubercle bacillus alone can and does cause inflammatory changes simulating the acute forms of pneumonia. Birch-Hirschfeld[1]: "Cheesy broncho-pneumonia cannot be considered a concurrent disease, for it is the primary effect of the tubercular infection, though it may not, and often does not, show itself in chronic phthisis until after the interstitial, or, more particularly, peri-bronchial changes have taken place. The cases in which acute diffuse desquamative pneumonia occur in areas of lung tissue, the seat of tuberculous disease, are quite different. Here the changes may take place during any stage of pulmonary tuberculosis. If the latter has been latent, we get the characteristic picture of an acute pneumonia; if in conjunction with a tuberculosis already recognized, but slowly advancing, we would speak of the complication as 'intercurrent pneumonia.'" Not infrequently it happens that in far advanced pulmonary tuberculosis of the apices, or in a tuberculous process of the base of the lung, an acute desquamative pneumonia attacks the remaining portions of the healthy lung, and death follows in a short time. Both the lobular and desquamative pneumonia, in such cases, are the direct product of tubercular infection. The influence of added pathogenic germs upon such processes must of necessity modify

1. Birch-Hirschfeld. *Pathologische Anatomie*, Vol. II., p. 430.

very materially the primary disease. The many clinical evidences of acute inflammatory changes in the lung in cases of chronic phthisis, with, not infrequently, cases in which physical signs of such exacerbation disappear entirely, are adequate to excite our curiosity for a careful study of the causes sufficiently potent to produce these effects.

Babes,[1] in 1888, found in fifty-two tuberculous children, forty-two cases in which other bacteria were found with the tubercle bacilli. The most common germ was the streptococcus pyogenes, while often the pneumococcus of Fränkel with other germs were present. The tubercle bacillus being, according to Baumgarten,[2] non-pyogenic, the association of these germs may account for the widespread and serious complications which we so often find beyond the seat of the tubercular lesion. Clinical and microscopic examination make positive the fact that the tubercle bacillus is confined to the seat of tubercular lesions, while the pyogenic germs admixed are widely spread in the various organs of the body. In adults, (Babes)[3] the association of other germs with the tubercle bacillus is perhaps less frequent, but in the cavities in pulmonary tuberculosis the association of the staphylococcus pyogenes aureus and a streptococcus were found. In nearly all tubercular lungs (Babes) the micrococcus, lanceolatus and streptococcus pyogenes are present. A fact of the greatest importance in conjunction with this subject is, that the administration of tuberculin to animals made them susceptible to the streptococci, pneumococci and other disease-producing germs which had ceased to be active or virulent in healthy animals.

Kitasato[4] found, in examining the secretions in tuberculous subjects from the deeper air-passages, after repeatedly washing in distilled water, large numbers of germs associated with tubercle bacilli, which he considered instrumental in producing many of the manifestations of the disease. Cornet,[5] after a large number of investigations, concluded that pulmonary tuberculosis is very likely to be a mixed infection, that the cavities and sputum show, in con-

1. Babes. *Sur les Associations Bacteriennes de la Tuberculose, Congress pour l'etude de la Tuberculose*, 1888.

2. Baumgarten. Quoted by Fränkel and Troje. *Zeitschrift für Klinische Medizin, Bd.* 24.

3. Babes. *Sur les Associations Bacteriennes de la Tuberculose, Congress pour l'etude de la Tuberculose*, 1891.

4. Kitasato. *Zeitschrift für Hygiene, Bd.* XI., p. 441.

5. Cornet. *Mischinfection der Lungen Tuberculose. Wiener Med. Wochenschrift, S.* 19–20, 1892.

junction with the tubercle bacillus, a rich addition of streptococci and staphylococci, less frequently bacilli. The first two, streptococci and staphylococci, are the most important from a pathogenic standpoint. Ortner,[1] after considerable observation in a series of cases of pulmonary tuberculosis, associated with pneumonic processes, concludes that there are two pathologic processes in pulmonary tuberculosis,—one leading to the formation of tubercle, the other giving rise to pneumonic infiltration.

These, he claims, differ both histologically and etiologically, tubercle causing the tubercle bacillus, the pneumococci causing the diffuse pneumonic changes. This may be and probably is a too positive statement, for the observation and experiments of Baumgarten,[2] Fränkel [3] and Troje, as well as Prudden, [4] prove, as already stated above, that the tubercle bacillus alone may cause a broncho-pneumonia, cheesy degeneration and other changes of an exudative and productive character.

It is interesting to note the arguments which Fränkel and Troje advance against the assumption that a mixed infection is instrumental in causing the pneumonic infiltration in these cases of pulmonary tuberculosis. The fact that the presence of other germs than the tubercle bacillus are found in the sputum, prompts the statement that the disease-producing agents are likely to be added to the sputum from the mouth, where, even in healthy individuals, pneumococci are present. The authors just quoted insist upon culture experiments as yielding the only conclusive proof of the pathogenic importance of these micrococci. They lay great stress upon the importance of distant tuberculous pneumonias and other pulmonary changes as being a direct result of the action of a diffusible poison elaborated by the tubercle bacillus. The recent experiments of Prudden in this connection confirm my original statement regarding the direct action of the tubercle bacillus. On the other hand, the conclusion is justified that an exudative pneumonia of varying extent and of extreme significance may occur in lungs which are the seat of tuberculous disease, as the result of secondary infection with other germs. (Prudden, Birch-Hirschfeld, Cornet, etc.)

You will bear with me while I call to your attention the leading features of the various forms of mixed infection as I have

1. Ortner. *Die Lungen Tuberculose als Mischinfection. Wien*, 1893.

2. Baumgarten. *Zeitschrift Klin. Medizin, Bd.* X., 188[illegible].

3. Fränkel and Troje. *Zeitschrift für Klin. Medizin, Bd.* XXIV., *Heft* 1, 2, 3, 4, 1893.

4. Prudden. New York *Medical Journal*, July 7, 1894.

chanced to meet them at the bedside, in association with pulmonary tuberculosis.

I will classify the cases as follows :

1. Cases of acute fibrinous pneumonia, in which the disease attacks an area of lung tissue, the greater part of which is the seat of infiltrating, but latent, tuberculosis. The previous history includes, as a rule, disease in a distant organ, from which pulmonary tuberculosis took its origin or with which it was coincident. The latent pulmonary deposit, as a rule, did not give rise to subjective symptoms before the advent of the acute pneumonia.

2. (*a*) Cases in which there is an acute croupous or catarrhal pneumonia in the immediate vicinity of tuberculous areas, the latter previously recognized, with changes in the infiltrated areas, usually at the apex ; the fibrinous disease running its course and terminating by crisis or lysis. The disease is not associated with hemoptysis as a prodromal or initial symptom. (*b*) Cases of chronic or subacute pulmonary tuberculosis in which acute catarrhal or croupous pneumonia attacks distant areas of the diseased or opposite lung, in which there is no early hemoptysis, but the tuberculous process is actively progressive, with physical signs of beginning or already completed disorganization.

3. Cases which may be called streptococcus pneumonia, in which the disease is added to either a latent or active pulmonary tuberculosis. Hemoptysis is present, usually, during the early stage of the acute exacerbation or immediately precedes the pneumonia. Here the complication depends largely upon the aspiration of infecting agents from the seat of the original infiltration and ultimate disorganization.

4. Cases of acute broncho-pneumonia, occasionally fibrinous, concurrent bacillary infection, where, as a result of lowered vitality, resulting usually from child-bearing, alcoholism, or unfavorable environment, there is in a comparatively short time rapid disorganization of lung tissue, ultimate cheesy infiltration, with the clinical evidences of coagulation necrosis, hectic fever and death.

The most unique and at the same time surprising cases of mixed infection which have come to my notice, are those in which there has been no suspicion of existing pulmonary tuberculosis antedating the accompanying pneumonia. There were no lung symptoms until the violent outbreak of croupous disease, and in no case which I have seen had the physician been consulted to prescribe for the patient or examine the lung physically. The general appearance of the patient had, in many cases, been so good that during the early days of the acute pneumonia no suspicion of the true state of affairs was entertained, and not until it became plain that the pneumonia was not following a typical course and bacteriological examinations were made, were the facts established which made it positive that we were dealing with mixed infection. In the majority of these cases the added element gave rise to a croupous pneumonia, which involved the area of latent disease and the tissues immediately adjacent to it.

While there are in many of these cases no subjective complaints or objective symptoms of pulmonary tuberculosis before the appearance of pneumonia, careful inquiry and a thorough search reveal the fact that there have been foci in distant organs, from which, in the light of later developments, pulmonary tuberculosis proceeded, or with which it was originally closely related. Many of these patients have good family histories, while their records strengthen the conclusion that lung tuberculosis may be present, but dormant, awaiting the advent of some depressing agent or added pulmonary disturbance. In other words, lower by the addition of the second germ the resisting power of the patient who has an unsuspected tuberculosis, and, as a rule, the result will be tissue disorganization and consequent progression of the original disease. I know that this conclusion is contrary to the belief of many who attribute to mixed infection little influence on latent tuberculosis, and who prognosticate favorably in cases of this class, but I give it as being in accord with my own clinical experience. The following case, hurriedly recited, exemplifies very clearly and I think with sufficient data the truth of the statements made, and gives positive proof of the fact, that croupous pneumonia can attack lung tissue the seat of tubercular disease:

E. C. A., male, merchant, aged 36, denies specific disease ; was seen first on June 21, 1892. His family history was negative, father and mother both living, in good health ; nowhere in the patient's family has there ever been anything like tuberculous disease; in fact, there had been no deaths in patient's immediate family. The patient was an intelligent man who had lived an exemplary life, was temperate in the true sense of the word, had never had any but the usual diseases of childhood. Since adolescence, had been well until about April, 1891, when after a number of weeks of pain and swelling in the right ischio-rectal fossa, a physician was consulted and found him suffering from an ischio-rectal abscess, for which an operation was ultimately performed, when the surgeon pronounced the disease tubercular. After four weeks the abscess and sinuses were completely healed, and patient appeared well. He resumed his position, which was one of great responsibility and care. His wife, an acute observer, unusually watchful, having abundant opportunity to note the advent of disease and symptoms if there were any, says that there was nothing so far as she could detect to cause her or her husband to believe that he was not in perfect health. After the healing of the abscess until the advent of the disease for which I was now consulted, the patient had never coughed nor had any respiratory disease or symptoms of which he was conscious, prior to June 19, 1892, or about twenty-four hours before my connection with the case. In fact, after the operation for the fistula and until the day when his acute symptoms commenced, he had gained in weight and strength believing himself all the time to be, as he said, "sound as a bullet." On June 19th, he was suddenly seized with a severe chill, pain in the upper regions of the right lung on inspiration, and a cough, which, when I saw him, was accompanied with a light yellow viscid expectoration. Immediately after his chill he had, as he expressed it, "a raging fever." When I saw him at 11 o'clock in the evening of the second day of his illness, I found him an able-bodied man, well nourished, with face flushed, skin dry, and temperature, 104°; pulse, 110°; respiration, 38–42. Examination of chest showed flatness marked at the right apex, losing itself in a small area of dulness below and final normal resonance. Careful percussion gave a normal percussion note over all parts of both lungs with the exception of the right infra and supra clavicular regions. At the right apex there was increased vocal fremitus. Auscultation elicited bronchial breathing, crepitant râles in abundance over area of dulness and flatness. There was also bronchophony. In short, we had in this case all the signs and symptoms of an acute croupous pneumonia of the right apex with a flatness so absolute, in conjunction with the unusual seat of the disease, as to give rise to suspicion sufficient to call for great care in the process of differentiation. Therefore, with a rusty expectoration on the second day of my attendance, a careful bacteriological examination of the sputum was made for both tubercle bacilli and pneumococci. The result of the examination with the microscope was positive. There were abundant pneumococci of Fränkel and Friedlander, with fields rich in streptococci, but no tubercle bacilli. Until the seventh day the clinical course corresponded in every respect with that of an ordinary croupous pneumonia, though the absolute flatness persisted unchanged. On the sixth day of my attendance the temperature fell to 99° F., with a profuse perspiration, remaining between that figure and 101° F. all day. On the following day, though the temperature reached 101°, there was no increase in the physical signs; the symptoms approximated those of the end stage of croupous pneumonia with, however, a persistence of bronchial breathing and small râles. After this day the temperature never again returned to the normal, there were large and coarse râles disseminated over both lungs, without much change in breath sounds or percussion note. The right apex failed to give satisfactory evidence of resolution. The sputum, still occasionally tinged with blood, was fast becoming purulent. Prompted by the persistent flatness and continued temperature ranging from 102–103° F., repeated microscopic examinations were made from day to day, when it was found that we were not only dealing with an acute croupous pneumonia, as shown by the presence of pneumococci, but there were now isolated tubercle bacilli to be found throughout the field. As the disease made advances it was evident that the lung tissue at the right apex was undergoing disorganization; small cavities had formed before the twentieth day. The patient on August 1st was turned over to Dr. Loomis, as I left for my vacation, when we were both satisfied that he was fast progressing toward the end, as the result of infiltrating tuberculosis and tubercular pneumonia.

This latter diagnosis we made at this stage owing to the fact that the microscope justified it, as did the symptoms also. On my return from Europe early in October, the patient was still alive, but disorganization had taken place in both lungs, the right apex was completely converted into cavities, while he had every appearance of the last stage of acute phthisis. His death followed about fifteen weeks after the beginning of the illness. We were unable to get an autopsy. This is indeed a unique and instructive case, in which there are many points of great practical value and interest.

The points of interest are:

1. The preceding tuberculosis of the ischio-rectal fossa.

2. The long period of perfect health following the abscess without subjective symptoms of lung involvement.

3. The undoubted croupous pneumonia of the right apex in an area of lung tissue, evidently flat as the result of latent tuberculous disease, the presence of such absolute flatness arousing suspicion.

4. The positive diagnosis of croupous pneumonia made as a result of microscopic examination; with an unusually clean history of the disease.

5. The evident termination of the pneumonia by crisis on the sixth day, and the ultimate return of the fever with evidences of tuberculous disease.

6. The absence of the tubercle bacilli until disorganization of degeneration of the infiltrate had taken place.

7. The evidences of streptococcus and tuberculous pneumonia after the croupous pneumonia had run its course, with changes in both lungs of a rapidly destructive character.

In this case our combined methods of physical and bacteriological examination made it clear that we were dealing with a croupous pneumonia which attacked an area of lung tissue already the seat of a latent and infiltrating tuberculosis. Whether this undetected tuberculous herd antedated the ischio-rectal infection, was cöincident with it, or resulted from it as a secondary infection, no one will attempt to establish. The croupus process, thoroughly typical and characteristic, certainly had a material influence in lighting the dormant tuberculous infection with which it was concurrent into activity.

Kinnicut[1] reported a similar case in 1894, commenting on its unique character. I have found that many authorities admit the possibility of such concurrent infection with latent tuberculosis. The differential diagnosis in such cases of mixed infection as the one just described, is ofttimes difficult and many authors claim almost impossible to make. This is particularly true of cheesy tuberculous pneumonia. Traube, whom I have already quoted, taught that tuberculous pneumonia is not ushered in with decided chills, while, as in our case, croupous pneumonia, when concurrent with latent pulmonary tuberculosis, begins with the severe chill of the ordinary uncomplicated disease.

In these cases of croupous pneumonia, bronchial breathing is an early symptom, while in acute cheesy pneumonia it comes on late. The crepitant râles of fibrinous concurrent pneumonia are as characteristic as are those of the unmixed cases. The presence of absolute flatness early in the disease, with an anomalous seat of the croupous process, must excite suspicion, though only in association with other symptoms. The return of persistent fever after the fall of the temperature as in crisis or lysis, must at once lead the clinician to conclude that there is an unusual complication, and in all probability tuberculosis is co-present. With symptoms of croupous pneumonia in any part of the lung and persistent fever, without the fall of the crisis or lysis, after from seven to ten days, the chances strongly favor the diagnosis of tuberculous pneumonia.

I firmly believe, as the result of a considerable experience with all forms of tuberculosis, that the failure to find tubercle bacilli in the sputum or secretions does not justify us in concluding that the disease is not tuberculosis. Patients without ulceration may yet

---

1. Kinnicut. *Medical Record*, October 11, 1894.

have far-reaching infiltrating or disseminated miliary tuberculosis and no bacilli in the sputum or bronchi. Such cases of miliary tuberculosis are frequent. Not until the cheesy infiltrate undergoes disorganization are we likely to find tubercle bacilli in the sputum. Bacteriological investigations revealing the presence of characteristic bacterial contamination, as in the case reported, make the diagnosis positive. It is necessary in these cases to make repeated bacteriological examinations.

The centrifuge must serve as a valuable adjuvant and will aid in clearing many of the doubtful cases.

The presence of pus and fibrine in conjunction with pneumococci, or even staphylococci, must lead at once to the conclusion that we are not dealing with an ordinary uncomplicated cheesy pneumonia. The clinician who depends for a positive diagnosis in cases of concurrent infection on subjective symptoms and physical signs alone, will make only uncertain diagnoses. Careful examination of the sputum, with a consideration of all that the case offers, from day to day, can alone interpret the true meaning of the symptoms of acute infection.

2. In the second type of mixed infection which I am attempting to establish, the previous history with physical signs and bacteriological examinations make positive the presence of a moderately active pulmonary tuberculosis or a latent infection with well-marked but limited changes in the lung. As a rule there are small cavities, and these either at the apex or near it. Most of these patients have consulted the physician before the advent of added infection, and in almost all tubercle bacilli were demonstrated with more or less cough before the acute exacerbation. The general condition early in some of these cases is not bad. There is no hemoptysis early in the acute disease, neither is the latter connected in any way with the ultimate pneumonia. The concurrent infection shows itself usually with well-marked acute symptoms. A more or less severe chill ushers in the disease, cough follows with characteristic rusty sputum containing not only tubercle bacilli but also pneumococci of Fränkel and Friedlander, streptococci and staphylococci, with pus fibrin or epithelial elements and occasional elastic fibers, varying according to the character of the inflammation. Following the chill there is a temperature which continues between 102° and 104° F., until about the sixth or seventh day, when there is a decided abatement of all the acute symptoms, or the temperature may gradually fall as in lysis to remain at about the level held before the complication. The added physical signs are characteristic either of lobar or lobular pneumonia.

2. (*a*) The process is most likely to be active in the previously healthy tissue immediately surrounding the old tuberculous deposit if the changes are limited or latent, or if there be only moderate disorganization of lung tissue. (*b*) If the preceding tuberculosis has been of a chronic or sub-acute character, and there is an active and progressive degeneration with considerable cavity formation, the acute exacerbation will attack, as a rule, the more dependent parts of the involved or healthy lung by the aspiration of infecting material from the seat of the original deposit or cavity.

In the cases belonging to subdivision *a* of this class, the pneumonic process may lead to a crisis or terminate by lysis ; the involved area of lung tissue becoming normal, the tuberculous process is but little influenced by the concurrent pneumonia.

On the other hand, in the cases belonging to subdivision *b* of this class, in which the acute changes are distant from the original seat of the tuberculous disease, the disease is either from the first a cheesy or tuberculous pneumonia or becomes so before the process has continued for any length of time. Disorganization is rapid ; the end is materially hastened by the complication of added infection. The microscopic picture in this class of cases is convincing.

3. In the majority of cases belonging to this class, the previous history will establish the fact that there has been either a latent tuberculosis with or without marked evidences of disorganization and cavity formation, a cheesy infiltrate which has not yet led to advanced tissue change, a central cavity, the walls of which may be guarded by a fibrous coat holding pure pus, a bloody serum with few shreds of necrotic tissue, or the disease may be in an actively progressive stage with rapid melting away of lung substance. With such changes, and the usual symptoms accompanying, tubercle bacilli abundantly present in the sputum, there is a sudden profuse hemoptysis, the temperature is in the course of from twenty-four to forty-eight hours elevated from one to three degrees above the level of that preceding the hemorrhage, the pulse is correspondingly accelerated, while the physical signs, rather tardily, give evidence of an acute broncho-pneumonia in parts distant from the seat of the original tuberculous deposit. In these cases there are usually only the evidences of a catarrhal process disseminated, with crepitant râles, a modified tympanitic dulness, but sometimes no change in percussion note. There is great dyspnea early after the hemorrhage. The fever is likely to rise with increasing evidences of cerebral involvement and profound infection, hemoptysis continues as a rule, with increasing weakness, death ending the scene within from seven to ten days after the beginning of the acute symptoms.

This may be the course of cases in which the patient to all appearances was in perfect health before the hemoptysis, and in which tuberculosis had never been suspected.

Not all cases of this class end with such acute symptoms or with evidences of such profound infection. It has not infrequently happened in my experience that the acute inflammatory changes in distant parts of the lung undergo absorption, and the patient, after the acute stage has passed, remains with the latent deposit or cavity unchanged. Our cases are less likely to have such persistent hemoptysis as was present in the cases reported by Baumler.[1]

Microscopic examination of the sputum and blood in these cases must be repeatedly made, when it will be found that the blood which has been aspirated into distant parts of the lung carries with it bacterial life, a preponderance of staphylococci and streptococci; tubercle bacilli, though present, are not in the ascendancy. It is this contamination from an original tuberculous focus, with the added elements capable of producing inflammatory and tissue changes, characteristic of lobar or lobular pneumonia, which is so often present after hemoptysis. (Birch-Hirschfeld,[2] Ziegler,[3] Cornet.[4])

The question of whether hemoptysis *per se* leads to tuberculosis, unless it be of tuberculous origin, is one which the bacteriologists of today, aided by the methods of precision which can be employed by every painstaking clinician, can answer without hesitation. It may be accepted as true, that hemoptysis associated with other symptoms of disturbed pulmonary function is of tuberculous origin, that when it is followed by phthisis immediately, it is originally an expression of existing tuberculous disorganization. In these cases of mixed infection which we are considering, where

---

1. Baumler. L. C., 1893.

2. Birch-Hirschfeld. *Lehrbuch der Pathologischer Anatomie, 3te Auflage, Leipzig,* 1887, *Bd.* II., p. 449.

3. Ziegler. *Lehrbuch der Allgemeine und Special Pathologische Anatomie, 7te Auflage,* Jena, 1892. *Bd.* 14, *S.* 697.

4. Cornet. *Ueber Mischinfection der Lungen Tuberculose.* XI. *Congress für Innere Medizin Verhandlungen,* p. 425.

added contamination is positive, and there is ultimately a second latent period of the disease, it becomes of the greatest practical importance to act promptly upon the theory that all forms of hemoptysis, not traumatic or associated with disease of the heart or large vessels, are tubercular. Inability to detect physical signs of a convincing character need not interfere with the safer conclusion, for if it be incorrect, it will be so only in very exceptional cases.

The benefit which will accrue to suffering humanity from such reasoning, with the rational therapeutic deductions following, can only be estimated when we consider the number of cases in which hemoptysis is one of the earliest symptoms of tubercular disease. The rapidly ending cases formerly included under the head of "Phthisis ab hemoptoe" were undoubtedly examples of mixed infection with lobar or lobular pneumonia and ultimate cheesy degeneration of original tuberculous herds.

4. In this, the last class to which I call your attention, there may be a clear history of previous active pulmonary tuberculosis which has become stationary or latent for a time, or, as happens in some of these cases, there may have been the previous history of a circumscribed pulmonary tuberculosis, with an ultimate pleurisy or empyema, after which the lung disease is held for a time in abeyance. The renewal of symptoms follows upon general depression or lowered vitality, ofttimes associated with child-birth, excesses of any kind, particularly in the use of alcohol and unfavorable environment. The clinical picture, during the early days of the concurrent disease, is that of an acute broncho-pneumonia, rarely fibrinous, while the number of attending symptoms and physical signs are usually characteristic of the uncomplicated non-tuberculous disease. The rapidity with which islands of lung tissue are involved in this broncho-pneumonic process is surprising, while the exhaustion of the patient is considerably unique. The pulse, particularly in women, with this complication at once becomes rapid and remains so during the entire course of the disease. The temperature is, as a rule, but little below 103° F., and usually above that point, while after the fourth or fifth day of the disease there are occasional morning remissions with evening exacerbations and profuse perspiration during the night.

When this stage is reached, it soon becomes evident, as a rule, that the disease is not to terminate with the broncho-pneumonia, for the tuberculous process is likely to gain the ascendancy, and the case becomes one of acute phthisis, terminating with all the clinical evidences of tissue disorganization and disseminated tuberculosis. In not a few cases the broncho-pneumonia is cleared, while the lung tissue originally involved in the tuberculous process at once shows the changes characteristic of acute consumption. The sputum does not always show tubercle bacilli until the acute broncho-pneumonia has continued for a few days. The predominating bacteria of the first few days of the disease are streptococci-staphylococci, with Friedlander pneumococci.

The Fränkel pneumococci is not usually present.

After the seventh day the tubercle bacillus is found with ease. The centrifugalized sputum during the early days has failed, as a rule, unless the tubercular process was active at the time of the broncho-pneumonia to show more than an occasional tubercle bacillus. It would be only natural to conclude that the acute pneumonia results in these cases from the co-presence of the bacteria above mentioned, in the tuberculous area and their ultimate dissemination to distant parts of both lungs by aspiration or through the usual vascular channels. But few of the cases fall back into a latent stage, as most patients die in the course of a few weeks or months.

### CONCLUSIONS.

1. Tubercle bacilli can, without the presence of added bacterial infection, cause changes in the lung, giving rise to symptoms of acute pneumonia in chronic or latent phthisis, which cannot be differentiated without bacteriological examination from non-tuberculous pneumonia.

2. Concurrent infection in tubercular phthisis modifies very materially the course of the disease, giving rise to many acute exacerbations and anatomical changes, hence the conclusion is justified that the clinical picture of the disease is, as a rule, complex. The double infection must be taken into account when indications for treatment are considered.

3. An acute croupous pneumonia can attack lung tissue, the seat of tubercular infiltration, or run its course in any part of the healthy or diseased lung. As a rule, the tuberculosis, if latent, is lighted into activity.

4. Aspiration from cavities of streptococci and other bacteria may give rise to acute pneumonia. This may be termed streptococcus pneumonia. Early and profuse hemoptysis is present in the majority of these cases.

5. Mixed infection in pulmonary tuberculosis is an important factor in lowering vitality and the resistance of the patient; all septic and pyemic processes arising in the course of tuberculous disease, may be traced to it.

6. The differential diagnosis of tuberculous pneumonia is ofttimes made with great difficulty. In all cases, bacteriological examination must be made repeatedly and culture experiments add to the refinement of such diagnoses.

Without repeated microscopic and bacteriological examination the physician must fail to interpret the pathological significance of the innumerable exacerbations of pulmonary tuberculosis. With such examinations repeatedly and conscientiously made, he will learn to recognize early the importance of mixed infection, and by immediate action, will be able to prolong the lives of these unfortunate patients, while in not a few cases ultimate cure will reward his efforts.

